# Liquid biopsies for early detection of gliomas: are we near a breakthrough?

**DOI:** 10.1097/MS9.0000000000003987

**Published:** 2025-09-30

**Authors:** Asra Amjad, Mir Raza Ali, Muddassir Khalid, Fred Segawa

**Affiliations:** a Department of Medicine, Islamic International Medical College Rawalpindi Pakistan, Riphah International University; b Department of Medicine, Jinnah Postgraduate Medical Centre, Karachi, Sindh, Pakistan; c Department of Medicine, Nishtar Medical University, Multan, Pakistan; d Department of Medicine, Makerere University School of Health Sciences, Kampala, Central Region, Uganda

*Dear Editor,*

Gliomas continue to be among the most aggressive and fatal CNS tumors, and their late discovery significantly restricts treatment alternatives, rendering the development of minimally invasive, high-precision diagnostic instruments a critical therapeutic imperative. Brain cancer across all-grade gliomas was correctly identified by machine learning analysis of plasma circulating free DNA (cfDNA) fragmentome characteristics (AUC = 0.90; 95% confidence interval, 0.87–0.93), and this finding was confirmed in a separate cohort. A new noninvasive detection method was made possible by cfDNA alterations that represented both tumor-derived fragmentation and changed white blood cell populations^[1]^.

Although these findings demonstrate the potential of plasma-based analysis, additional research indicates that cerebrospinal fluid (CSF) might provide even more precise diagnosis. While plasma ctDNA detection was only 14% of glioma patients, CSF-ctDNA was found in 59% of them with 84% tumor-CSF mutation concordance. A considerably worse survival rate was associated with a higher frequency of ctDNA variant alleles, demonstrating the validity of CSF as a source for glioma mutational profiling^[2]^. The necessity of carefully choosing the sampling medium to optimize sensitivity and prognostic value is further supported by these biological variations. AI-driven algorithms, liquid biopsies [ctDNA, extracellular vesicles (EVs), circulating tumor cells (CTCs), and tumor-educated platelets], and advanced imaging (diffusion tensor imaging, magnetic resonance spectroscopy, and new positron emission tomography tracers) are improving the accuracy of CNS tumor diagnosis and therapy monitoring. Although there are still issues with accessibility and standardization, integrating these techniques offers better outcomes and personalization in neuro-oncology^[3]^. Tracking tumor changes throughout time, rather than just at diagnosis, is also made possible by this integration. With 87.2% of samples producing enough DNA, longitudinal cerebral CSF cfDNA profiling was possible in glioma patients. With a correlation to treatment success and the opportunity for dynamic disease monitoring, tumor-associated variant allele frequency and copy-number burden decreased during chemoradiation and increased throughout progression^[4]^.

When taken into account in conjunction with the underlying biology of glioblastoma advancement, this monitoring capability becomes even more significant. Key oncogenic pathways [epidermal growth factor receptor, platelet-derived growth factor receptor, and phosphatidylinositol-3-kinase/protein kinase B/mammalian target of rapamycin pathway (pi3k/AKT/Mtor)], epigenetic changes, and an immunosuppressive microenvironment are what propel the evolution of GBM. Together with precision medicine, emerging molecular, immunological, metabolic, and locoregional medicines provide interdisciplinary approaches to improve quality of life, increase survival, and overcome resistance^[5]^. The growing therapeutic toolset increases the need for precise and prompt biomarker detection. By identifying ctDNA, CTCs, EVs, miRNAs, proteins, and metabolites that penetrate the blood–brain barrier, liquid biopsy utilizing blood or CSF provides a minimally invasive method of diagnosing and tracking GBM. Although promising, methodological variability and small cohorts limit its clinical utility, making it best suited as a supplemental technique to tissue biopsy to improve monitoring and diagnostic accuracy^[6]^.

Extracellular vesicles are notable within this spectrum as both high-value diagnostic carriers and functional drivers of illness. In GBM, extracellular vesicles function as a promising liquid biopsy tool that allows for precise evaluation of disease severity and progression beyond the scope of traditional diagnostics, in addition to driving tumor progression by forming a heterogeneous microenvironment^[7]^. Specialized assays that identify molecular targets that are clinically relevant enhance this diagnostic capacity even more. The maximum reported sensitivity of 85.7% was achieved by sEV-based liquid biopsy in detecting methylated O6-methylguanine-DNA methyltransferase in isocitrate dehydrogenase—wild-type GB, demonstrating significant agreement with tissue analysis. With major ramifications for enhancing prognostic and treatment approaches, this method provides a potent instrument for tracking the course of the disease and resolving molecular underdetection^[8]^.

The range of observable changes is further supported by parallel work in CSF cfDNA analysis. The BBB makes blood-based detection difficult; however, CSF-based cfDNA analysis provides a less invasive and efficient method for identifying tumor-specific genetic changes in gliomas. NGS and digital PCR have shown promise in successful experiments; however, there are still technical and standardization issues^[9]^.

Meanwhile, plasma-based methods keep offering fresh perspectives. Mutations were found in 93.8% of samples by plasma ctDNA analysis in gliomas, with 25% of the samples being found only in plasma. Following temozolomide, new mutations in the mismatch repair gene [MutS Homolog 2 (MSH2) and MSH6] appeared and frequently appeared before being discovered in recurring tumor tissue, indicating the possibility of early resistance monitoring and tissue biopsy supplementation^[10]^.

When combined, these developments establish liquid biopsy, specifically, CSF-based cfDNA and sEV platforms, as a crucial advancement in the detection and tracking of gliomas. In order to make early glioma identification a commonplace procedure rather than a rare discovery, the next step is to scale, standardize, and incorporate these techniques into clinical workflows.

This manuscript complies with TITAN guidelines, 2025, declaring no use of AI^[11]^.

## Data Availability

Data can be made available on a reasonable request.
